# Supplementary material for: An overview of the osseous palmar sesamoid in Anura, with the particular case of some Rhinella species
Source: PeerJ. 2023 May 15;11:e15063. doi: 10.7717/peerj.15063 (PMC10194070; doi:10.7717/peerj.15063)
Supplement: File S1 [file peerj-11-15063-s001.docx]

**Material Repository**

**Dissection Material**

*Rhinella arenarum* FML 29863; FML 29859; DIAM 177, 178; L 935; LS 337B; Laura Ponssa personal collection (MLP s/d, 2 specimens)

*Rhinella henselii* CFBH 20277

*Rhinella major* FML 29837

*Rhinella dorbigni* DIAM 344

*Rhinella disptyca* DIAM 460

*Rhinella margaritifera* DIAM 514, DIAM 516, DIAM 517

**Morphosource Material**

|  | **Images** | **Link** | **MorphSource identifier** |
| --- | --- | --- | --- |
| 1 | CT scan of ***Alytes obstetricans*** CAS-SUA-21691 | https://www.morphosource.org/concern/media/000024008?locale=en | [doi:10.17602/M2/M24008](https://doi.org/10.17602/M2/M24008) |
| 2 | CT scan of KU-H-144217, ***Discoglossus pictus*** | https://www.morphosource.org/concern/media/000029728?locale=en | [ark:/87602/m4/M29728](http://n2t.net/ark:/87602/m4/M29728)  https://doi.org/10.17602/M2/M29728 |
| 3 | CT scan ku:kuh:31372 ***Ascaphus montanus*** | https://www.morphosource.org/concern/media/000038213?locale=en | [ark:/87602/m4/M38213](http://n2t.net/ark:/87602/m4/M38213)  https://doi.org/10.17602/M2/M38213 |
| 4 | Whole body STL CT of ***Ascaphus truei*** uf:herp:80664 | https://www.morphosource.org/concern/media/000011258?locale=en | [doi:10.17602/M2/M11258](https://doi.org/10.17602/M2/M11258) |
| 5 | STL of skeleton CT scan of ***Leiopelma hamiltoni*** CAS53931 | https://www.morphosource.org/concern/media/000024295?locale=en | [ark:/87602/m4/M24295](http://n2t.net/ark:/87602/m4/M24295)  https://doi.org/10.17602/M2/M24295 |
| 6 | Whole body STL CT Scan of ***Barbourula busuangensis*** | https://www.morphosource.org/concern/media/000011259?locale=en | [ark:/87602/m4/M11259](http://n2t.net/ark:/87602/m4/M11259)  https://doi.org/10.17602/M2/M11259 |
| 7 | CT Scan [uf:herp:96648 ***Bombina maxima***](https://www.morphosource.org/concern/biological_specimens/0000S3733) | https://www.morphosource.org/concern/media/000012422?locale=en | [ark:/87602/m4/M12422](http://n2t.net/ark:/87602/m4/M12422)  https://doi.org/10.17602/M2/M12422 |
| 8 | CT scan of ***Pipa parva*** [uf:herp:37924](https://www.morphosource.org/concern/biological_specimens/0000S3720) Full body STL | https://www.morphosource.org/concern/media/000012417?locale=en | [ark:/87602/m4/M12417](http://n2t.net/ark:/87602/m4/M12417)  https://doi.org/10.17602/M2/M12417 |
| 9 | Whole body ethanol preserved cas: herp:207765 ***Xenopus allofraseri*** | https://www.morphosource.org/concern/media/000058061?locale=en | [ark:/87602/m4/M58061](http://n2t.net/ark:/87602/m4/M58061)  https://doi.org/10.17602/M2/M58061 |
| 10 | CT scan of UF-H-92704, ***Xenopus clivii*** | https://www.morphosource.org/concern/media/000026263?locale=en | [ark:/87602/m4/M26263](http://n2t.net/ark:/87602/m4/M26263)  https://doi.org/10.17602/M2/M26263 |
| 11 | Whole body ethanol preserved  cas:herp:152778  ***Xenopus borealis*** | https://www.morphosource.org/concern/media/000058062?locale=en | [ark:/87602/m4/M58062](http://n2t.net/ark:/87602/m4/M58062)  https://doi.org/10.17602/M2/M58062 |
| 12 | CT scan of Spea multiplicata whole body STL uf:herp:100788 ***Spea multiplicata*** | https://www.morphosource.org/concern/media/000011079?locale=en | [ark:/87602/m4/M11079](http://n2t.net/ark:/87602/m4/M11079)  https://doi.org/10.17602/M2/M11079 |
| 13 | STL of skeleton Migrated MorphoSource 1 Media Group Title: CT scan of UF-H-9620, ***Scaphiopus holbrookii*** | https://www.morphosource.org/concern/media/000022641?locale=en | [doi:10.17602/M2/M22641](https://doi.org/10.17602/M2/M22641) |
| 14 | STL of Skeleton CT Scan of CAS-H-94059, ***Pelodytes caucasicus*** | https://www.morphosource.org/concern/media/000024580?locale=en | [ark:/87602/m4/M24580](http://n2t.net/ark:/87602/m4/M24580)  https://doi.org/10.17602/M2/M24580 |
| 15 | STL of skeleton CT Scan of UF-H-36935, ***Pelabtes fuscus*** | https://www.morphosource.org/concern/media/000024459?locale=en | [doi:10.17602/M2/M24459](https://doi.org/10.17602/M2/M24459) |
| 16 | STL of skeleton CT scan of CAS-H-92371, ***Pelobates varaldii*** | https://www.morphosource.org/concern/media/000025396?locale=en | [ark:/87602/m4/M25396](http://n2t.net/ark:/87602/m4/M25396)  https://doi.org/10.17602/M2/M25396 |
| 17 | Whole body CT scan of ***Brachytarsophrys carinensis*** | https://www.morphosource.org/concern/media/000016763?locale=en | [doi:10.17602/M2/M16763](https://doi.org/10.17602/M2/M16763) |
| 18 | STL of skeleton Migrated CT scan of ***Leptobranchium hasseltii*** | https://www.morphosource.org/concern/media/000016286?locale=en | [ark:/87602/m4/M16286](http://n2t.net/ark:/87602/m4/M16286)  https://doi.org/10.17602/M2/M16286 |
| 19 | CT scan of Skeleton of ***Xenophrys major*** cas:herp:245473 | https://www.morphosource.org/concern/media/000038055?locale=en | [ark:/87602/m4/M38055](http://n2t.net/ark:/87602/m4/M38055)  https://doi.org/10.17602/M2/M38055 |
| 20 | CT scan of uf:herp:100828  ***Hadromophryne natalensis*** | https://www.morphosource.org/concern/media/000012695?locale=en | [doi:10.17602/M2/M12695](https://doi.org/10.17602/M2/M12695) |
| 21 | Skeleton STL CT scan of cas-herp-156289, ***Hadromophryne natalensis*** | https://www.morphosource.org/concern/media/000033063?locale=en | [ark:/87602/m4/M33063](http://n2t.net/ark:/87602/m4/M33063)  https://doi.org/10.17602/M2/M33063 |
| 22 | STL of skeleton CT scan of CAS-H-10082, ***Caudiverbera caudiverbera*** | https://www.morphosource.org/concern/media/000022560?locale=en | [doi:10.17602/M2/M22560](https://doi.org/10.17602/M2/M22560) |
| 23 | Skeleton Mesh usnm:amphibians & reptiles:118774  ***Limnodynastes dumerili*** | https://www.morphosource.org/concern/media/000116397?locale=en | [ark:/87602/m4/M116397](http://n2t.net/ark:/87602/m4/M116397)  <https://doi.org/10.17602/M2/M116397> |
| 24 | Skeleton Mesh usnm:amphibians & reptiles:285080  ***Limnodynastes dorsalis*** | https://www.morphosource.org/concern/media/000116398?locale=en | [ark:/87602/m4/M116398](http://n2t.net/ark:/87602/m4/M116398)  <https://doi.org/10.17602/M2/M116398> |
| 25 | Skeleton Mesh specimens:mvz:herp:81531 ***Crinia signifera*** | https://www.morphosource.org/concern/media/000116392?locale=en | [ark:/87602/m4/M116392](http://n2t.net/ark:/87602/m4/M116392)  <https://doi.org/10.17602/M2/M116392> |
| 26 | STL of skeleton CT scan of UF-H-62022, ***Rhinoderma darwinii*** | https://www.morphosource.org/concern/media/000025059?locale=en | [ark:/87602/m4/M25059](http://n2t.net/ark:/87602/m4/M25059)  https://doi.org/10.17602/M2/M25059 |
| 27 | Skeleton Mesh usnm:amphibians & reptiles:64054  ***Mixophyes balbus*** | https://www.morphosource.org/concern/media/000116400?locale=en | [ark:/87602/m4/M116400](http://n2t.net/ark:/87602/m4/M116400)  <https://doi.org/10.17602/M2/M116400> |
| 28 | CT scan of body  of cm:herps:68395  ***Alsodes nodosus*** | https://www.morphosource.org/concern/media/000020871?locale=en | [ark:/87602/m4/M20871](http://n2t.net/ark:/87602/m4/M20871)  https://doi.org/10.17602/M2/M20871 |
| 29 | CT scan of body of cas:herp:141967  ***Atelognathus patagonicus*** | https://www.morphosource.org/concern/media/000021953?locale=en | [ark:/87602/m4/M21953](http://n2t.net/ark:/87602/m4/M21953)  https://doi.org/10.17602/M2/M21953 |
| 30 | CT scan of Skeleton of uf:herp:39734  ***Telmatobius thompsoni*** | https://www.morphosource.org/concern/media/000020345?locale=en | [ark:/87602/m4/M20345](http://n2t.net/ark:/87602/m4/M20345)  https://doi.org/10.17602/M2/M20345 |
| 31 | CT scan of body CT scan of cas:herp:85253  ***Batrachyla taeniata*** | https://www.morphosource.org/concern/media/000021960?locale=en | [ark:/87602/m4/M21960](http://n2t.net/ark:/87602/m4/M21960)  https://doi.org/10.17602/M2/M21960 |
| 32 | STL of skeleton CT scan of cas:herp:84998  ***Ceratophrys aurita*** | https://www.morphosource.org/concern/media/000016100?locale=en | [ark:/87602/m4/M16100](http://n2t.net/ark:/87602/m4/M16100)  https://doi.org/10.17602/M2/M16100 |
| 33 | CT scan of body uf:herp:12347  ***Lepidobatrachus laevis*** | https://www.morphosource.org/concern/media/000020355?locale=en | [ark:/87602/m4/M20355](http://n2t.net/ark:/87602/m4/M20355)  https://doi.org/10.17602/M2/M20355 |
| 34 | Skeleton STL CT scan of USNM92599, ***Cryptobatrachus boulengeri*** | https://www.morphosource.org/concern/media/000036084?locale=en | [ark:/87602/m4/M36084](http://n2t.net/ark:/87602/m4/M36084)  https://doi.org/10.17602/M2/M36084 |
| 35 | STL of skeleton whole body ethanol preserved uf:herp:30080  ***Gastrotheca dysprosita*** | https://www.morphosource.org/concern/media/000055496?locale=en | [ark:/87602/m4/M55496](http://n2t.net/ark:/87602/m4/M55496)  https://doi.org/10.17602/M2/M55496 |
| 36 | STL of skeleton whole body ethanol preserved uf:herp:42834  ***Gastrotheca longipes*** | https://www.morphosource.org/concern/media/000055499?locale=en | [ark:/87602/m4/M55499](http://n2t.net/ark:/87602/m4/M55499)  https://doi.org/10.17602/M2/M55499 |
| 37 | STL of skeleton CT scan of uf:herp:65783  ***Gastrotheca peruana*** | https://www.morphosource.org/concern/media/000032030?locale=en | [ark:/87602/m4/M32030](http://n2t.net/ark:/87602/m4/M32030)  https://doi.org/10.17602/M2/M32030 |
| 38 | CT scan of body uf:herp:65801  ***Gastrotheca peruana*** | https://www.morphosource.org/concern/media/000016281?locale=en | [doi:10.17602/M2/M16281](https://doi.org/10.17602/M2/M16281) |
| 39 | STL of skeleton Whole body ethanol preserved uf:herp:65801  ***Gastrotheca peruana*** | https://www.morphosource.org/concern/media/000055504?locale=en | [ark:/87602/m4/M55504](http://n2t.net/ark:/87602/m4/M55504)  https://doi.org/10.17602/M2/M55504 |
| 40 | STL skeleton amnh:herpetology: a-1342 ***Hemiphractus helioi*** | https://www.morphosource.org/concern/media/000078758?locale=en | [ark:/87602/m4/M78758](http://n2t.net/ark:/87602/m4/M78758)  https://doi.org/10.17602/M2/M78758 |
| 41 | STL of skeleton whole body ethanol preserved uf:herp:98224  ***Gastrotheca riobambae*** | https://www.morphosource.org/concern/media/000055510?locale=en | [ark:/87602/m4/M55510](http://n2t.net/ark:/87602/m4/M55510)  https://doi.org/10.17602/M2/M55510 |
| 42 | STL of body CT scan of zsm:36/0 ***Hemiphractus fasciatus*** | https://www.morphosource.org/concern/media/000018826?locale=en | [doi:10.17602/M2/M18826](https://doi.org/10.17602/M2/M18826) |
| 43 | STL of skeleton CT scan of uf:herp:43205  ***Hemiphractus proboscideus*** | https://www.morphosource.org/concern/media/000020026?locale=en | [ark:/87602/m4/M20026](http://n2t.net/ark:/87602/m4/M20026)  https://doi.org/10.17602/M2/M20026 |
| 44 | STL of body CT scan of KU167241, ***Stefania scalae*** | https://www.morphosource.org/concern/media/000037815?locale=en | [ark:/87602/m4/M37815](http://n2t.net/ark:/87602/m4/M37815)  https://doi.org/10.17602/M2/M37815 |
| 45 | CT scan of body uf:herp:67472 ***Acris crepitans*** | https://www.morphosource.org/concern/media/000020028?locale=en | [ark:/87602/m4/M20028](http://n2t.net/ark:/87602/m4/M20028)  https://doi.org/10.17602/M2/M20028 |
| 46 | CT scan of body uf:herp:67444 ***Acris crepitans*** | https://www.morphosource.org/concern/media/000019563?locale=en | [ark:/87602/m4/M19563](http://n2t.net/ark:/87602/m4/M19563)  https://doi.org/10.17602/M2/M19563 |
| 47 | STL of skeleton CT scan of uf:herp:107362  ***Acris gryllus*** | https://www.morphosource.org/concern/media/000020037?locale=en | [doi:10.17602/M2/M20037](https://doi.org/10.17602/M2/M20037) |
| 48 | CT scan of body of UF-H-137291, ***Agalychnis calcarifer*** | https://www.morphosource.org/concern/media/000026266?locale=en | [ark:/87602/m4/M26266](http://n2t.net/ark:/87602/m4/M26266)  https://doi.org/10.17602/M2/M26266 |
| 49 | STL CT scan, whole body uf:herp:137287  ***Anotheca spinosa*** | https://www.morphosource.org/concern/media/000011876?locale=en | [ark:/87602/m4/M11876](http://n2t.net/ark:/87602/m4/M11876)  https://doi.org/10.17602/M2/M11876 |
| 50 | STL of Skeleton CT scan of KU116935, ***Argenteohyla siemersi*** | https://www.morphosource.org/concern/media/000037687?locale=en | [ark:/87602/m4/M37687](http://n2t.net/ark:/87602/m4/M37687)  https://doi.org/10.17602/M2/M37687 |
| 51 | STL of Skeleton CT scan of KU92220, ***Corythomantis greeningi*** | https://www.morphosource.org/concern/media/000037680?locale=en | [ark:/87602/m4/M37680](http://n2t.net/ark:/87602/m4/M37680)  https://doi.org/10.17602/M2/M37680 |
| 52 | CT scan of body uf:herp:30274 ***Hyla andersonii*** | https://www.morphosource.org/concern/media/000012717?locale=en | [ark:/87602/m4/M12717](http://n2t.net/ark:/87602/m4/M12717)  https://doi.org/10.17602/M2/M12717 |
| 53 | CT scan of body uf:herp:64907 ***Hyla chrysoscelis*** | https://www.morphosource.org/concern/media/000020320?locale=en | [ark:/87602/m4/M20320](http://n2t.net/ark:/87602/m4/M20320)  https://doi.org/10.17602/M2/M20320 |
| 54 | CT scan of body uf:herp:123473 ***Hyla cinerea*** | https://www.morphosource.org/concern/media/000020330?locale=en | [ark:/87602/m4/M20330](http://n2t.net/ark:/87602/m4/M20330)  https://doi.org/10.17602/M2/M20330 |
| 55 | CT scan of body uf:herp:66557 ***Hyla femoralis*** | https://www.morphosource.org/concern/media/000013145?locale=en | [ark:/87602/m4/M13145](http://n2t.net/ark:/87602/m4/M13145)  https://doi.org/10.17602/M2/M13145 |
| 56 | CT volume and derivatives ku:kuh:30404  ***Ecnomiohyla miliaria*** | https://www.morphosource.org/concern/media/000065749?locale=en | [ark:/87602/m4/M65749](http://n2t.net/ark:/87602/m4/M65749)  https://doi.org/10.17602/M2/M65749 |
| 57 | Mesh Ct ummz:herps:118171  ***Hyla mixomaculata*** | https://www.morphosource.org/concern/media/000057248?locale=en | [ark:/87602/m4/M57248](http://n2t.net/ark:/87602/m4/M57248)  https://doi.org/10.17602/M2/M57248 |
| 58 | MicroCT volume and derivatives whole body ku:kuh:100899 ***Hyla nephila*** | https://www.morphosource.org/concern/media/000065743?locale=en | [ark:/87602/m4/M65743](http://n2t.net/ark:/87602/m4/M65743)  https://doi.org/10.17602/M2/M65743 |
| 59 | MicroCT volume and derivatives whole body ku:kuh:195550 ***Hyla salvaje*** | https://www.morphosource.org/concern/media/000065752?locale=en | [ark:/87602/m4/M65752](http://n2t.net/ark:/87602/m4/M65752)  https://doi.org/10.17602/M2/M65752 |
| 60 | CT skeleton ummz:herps:104779  ***Hyla smithi*** | https://www.morphosource.org/concern/media/000045199?locale=en | [ark:/87602/m4/M45199](http://n2t.net/ark:/87602/m4/M45199)  https://doi.org/10.17602/M2/M45199 |
| 61 | MicroCT volume and derivatives ku:kuh:95416  ***Ecnomiohyla valancifer*** | https://www.morphosource.org/concern/media/000065754?locale=en | [ark:/87602/m4/M65754](http://n2t.net/ark:/87602/m4/M65754)  https://doi.org/10.17602/M2/M65754 |
| 62 | CT scan of body uf:herp:73746  ***Hypsiboas boans*** | https://www.morphosource.org/concern/media/000020317?locale=en | [ark:/87602/m4/M20317](http://n2t.net/ark:/87602/m4/M20317)  https://doi.org/10.17602/M2/M20317 |
| 63 | STL of Skeleton CT Scan of UF-H-109124, ***Litoria aurea*** | https://www.morphosource.org/concern/media/000025349?locale=en | [ark:/87602/m4/M25349](http://n2t.net/ark:/87602/m4/M25349)  https://doi.org/10.17602/M2/M25349 |
| 64 | STL of body CT scan of ku-kuh-152476, ***Nyctimantis rugiceps*** | https://www.morphosource.org/concern/media/000037809?locale=en | [ark:/87602/m4/M37809](http://n2t.net/ark:/87602/m4/M37809)  https://doi.org/10.17602/M2/M37809 |
| 65 | STL of Skeleton CT scan of ***Crossodactylus trachystomus*** CM-H-2662 | https://www.morphosource.org/concern/media/000024014?locale=en | [ark:/87602/m4/M24014](http://n2t.net/ark:/87602/m4/M24014)  https://doi.org/10.17602/M2/M24014 |
| 66 | STL of body CT scan of USNM-amphibians & reptiles-81155, ***Crossodactylus gaudichaudii*** | https://www.morphosource.org/concern/media/000039539?locale=en | [ark:/87602/m4/M39539](http://n2t.net/ark:/87602/m4/M39539)  https://doi.org/10.17602/M2/M39539 |
| 67 | STL of skeleton CT scan of ***Acanthixalus spinosus*** CAS-H-153800 | https://www.morphosource.org/concern/media/000024233?locale=en | [ark:/87602/m4/M24233](http://n2t.net/ark:/87602/m4/M24233)  https://doi.org/10.17602/M2/M24233 |
| 68 | Skeleton STL for cas:herp:196702  ***Kassinula wittei*** | https://www.morphosource.org/concern/media/000040063?locale=en | [ark:/87602/m4/M40063](http://n2t.net/ark:/87602/m4/M40063)  https://doi.org/10.17602/M2/M40063 |
| 69 | Whole body STL CT scan of UF-H-107200, ***Ameerega trivittatus*** | https://www.morphosource.org/concern/media/000029052?locale=en | [ark:/87602/m4/M29052](http://n2t.net/ark:/87602/m4/M29052)  https://doi.org/10.17602/M2/M29052 |
| 70 | STL of skeleton CT scan of CAS-H-10670, ***Allobates kingsburyi*** | https://www.morphosource.org/concern/media/000024017?locale=en | [ark:/87602/m4/M24017](http://n2t.net/ark:/87602/m4/M24017)  https://doi.org/10.17602/M2/M24017 |
| 71 | Skeleton and skull STLs mcz:herp:a-91234  ***Colostethus latinasus*** | https://www.morphosource.org/concern/media/000115564?locale=en | [ark:/87602/m4/M115564](http://n2t.net/ark:/87602/m4/M115564)  https://doi.org/10.17602/M2/M115564 |
| 72 | Mesh skeleton CT of ummz:herps:167655  ***Andinobates minutus*** | https://www.morphosource.org/concern/media/000045192?locale=en | [ark:/87602/m4/M45192](http://n2t.net/ark:/87602/m4/M45192)  https://doi.org/10.17602/M2/M45192 |
| 73 | Whole body CT scan of UF-H-80874, ***Oophaga pumilio*** | https://www.morphosource.org/concern/media/000028400?locale=en | [ark:/87602/m4/M28400](http://n2t.net/ark:/87602/m4/M28400)  https://doi.org/10.17602/M2/M28400 |
| 74 | Whole body STL CT scan of UF-H-71742, ***Phyllobates bicolor*** | https://www.morphosource.org/concern/media/000028391?locale=en | [ark:/87602/m4/M28391](http://n2t.net/ark:/87602/m4/M28391)  https://doi.org/10.17602/M2/M28391 |
| 75 | Skeleton stl of ypm:vz:ypm hera 020210 ***Silverstoneia flotator*** | https://www.morphosource.org/concern/media/000115217?locale=en | [ark:/87602/m4/M115217](http://n2t.net/ark:/87602/m4/M115217)  https://doi.org/10.17602/M2/M115217 |
| 76 | STL of skeleton CT scan of mhnci:10274  ***Brachycephalus coloratus*** | https://www.morphosource.org/concern/media/000014777?locale=en | [ark:/87602/m4/M14777](http://n2t.net/ark:/87602/m4/M14777)  https://doi.org/10.17602/M2/M14777 |
| 77 | Whole body STL CT scan of mhnci:10295  ***Brachycephalus albolineatus*** | <https://www.morphosource.org/concern/media/000014777?locale=en>  WRONG LINK | [ark:/87602/m4/M14739](http://n2t.net/ark:/87602/m4/M14739)  https://doi.org/10.17602/M2/M14739 |
| 78 | CT scan of body CT scan of MHNCI-10285, ***Brachycephalus curupira*** Paratype | https://www.morphosource.org/concern/media/000014782?locale=en | [ark:/87602/m4/M14782](http://n2t.net/ark:/87602/m4/M14782)  https://doi.org/10.17602/M2/M14782 |
| 79 | CT scan of body CT scan of uf:herp:72725  ***Brachycephalus ephippium*** | https://www.morphosource.org/concern/media/000012426?locale=en | [doi:10.17602/M2/M12426](https://doi.org/10.17602/M2/M12426) |
| 80 | CT scan of body of usnm:amphibians & reptiles:96454  ***Ischnocnema gualteri*** | https://www.morphosource.org/concern/media/000022193?locale=en | [ark:/87602/m4/M22193](http://n2t.net/ark:/87602/m4/M22193)  https://doi.org/10.17602/M2/M22193 |
| 81 | STL of skeleton CT scan of CAS SU-A-11453, ***Oreobates quixensis*** | https://www.morphosource.org/concern/media/000022890?locale=en | [ark:/87602/m4/M22890](http://n2t.net/ark:/87602/m4/M22890)  https://doi.org/10.17602/M2/M22890 |
| 82 | CT scan of body CT scan of uf:herp:157233  ***Craugastor laticeps*** | https://www.morphosource.org/concern/media/000012670?locale=en | [ark:/87602/m4/M12670](http://n2t.net/ark:/87602/m4/M12670)  https://doi.org/10.17602/M2/M12670 |
| 83 | CT scan of body of ku:kuh:315000  ***Ceuthomantis smaragdinus*** | https://www.morphosource.org/concern/media/000024433?locale=en | [ark:/87602/m4/M24433](http://n2t.net/ark:/87602/m4/M24433)  https://doi.org/10.17602/M2/M24433 |
| 84 | STL of skeleton CT scan of uf:herp:68063  ***Barycholos pulcher*** | https://www.morphosource.org/concern/media/000020040?locale=en | [ark:/87602/m4/M20040](http://n2t.net/ark:/87602/m4/M20040)  https://doi.org/10.17602/M2/M20040 |
| 85 | STL of body CT scan of UF-H-68066, ***Barycholos pulcher*** | https://www.morphosource.org/concern/media/000026382?locale=en | [ark:/87602/m4/M26382](http://n2t.net/ark:/87602/m4/M26382)  https://doi.org/10.17602/M2/M26382 |
| 86 | CT scan of body CT Scab of uf:herp:40764  ***Hypodactylus araiodactylus*** | https://www.morphosource.org/concern/media/000023013?locale=en | [ark:/87602/m4/M23013](http://n2t.net/ark:/87602/m4/M23013)  https://doi.org/10.17602/M2/M23013 |
| 87 | STL of skeleton CT scan of ***Strabomantis anomalus*** CAS-H-119754 | https://www.morphosource.org/concern/media/000024021?locale=en | [ark:/87602/m4/M24021](http://n2t.net/ark:/87602/m4/M24021)  https://doi.org/10.17602/M2/M24021 |
| 88 | STL of Skeleton CT scan of CM-H-147828, ***Odontophrynus americanus*** | https://www.morphosource.org/concern/media/000025346?locale=en | [ark:/87602/m4/M25346](http://n2t.net/ark:/87602/m4/M25346)  https://doi.org/10.17602/M2/M25346 |
| 89 | whole body CT scan of CM-H-45986, ***Proceratophrys boiei*** | https://www.morphosource.org/concern/media/000029141?locale=en | [ark:/87602/m4/M29141](http://n2t.net/ark:/87602/m4/M29141)  https://doi.org/10.17602/M2/M29141 |
| 90 | CT scan of body CT scan of uf:herp:43260  ***Adenomera andreae*** | https://www.morphosource.org/concern/media/000013750?locale=en | [doi:10.17602/M2/M13750](https://doi.org/10.17602/M2/M13750) |
| 91 | STL of skeleton CT scan of uf-herp-103788, ***Leptodactylus pentadactylus*** | https://www.morphosource.org/concern/media/000050036?locale=en | [ark:/87602/m4/M50036](http://n2t.net/ark:/87602/m4/M50036)  https://doi.org/10.17602/M2/M50036 |
| 92 | CT scan of body CT scan of uf:herp:43260  ***Adenomera andreae*** | https://www.morphosource.org/concern/media/000013750?locale=en | [doi:10.17602/M2/M13750](https://doi.org/10.17602/M2/M13750) |
| 93 | STL of skeleton CT scan of uf-herp-103788, ***Leptodactylus pentadactylus*** | https://www.morphosource.org/concern/media/000050036?locale=en | [ark:/87602/m4/M50036](http://n2t.net/ark:/87602/m4/M50036)  https://doi.org/10.17602/M2/M50036 |
| 94 | CT scan of body CT scan of fmnh:amphibians and reptiles:1580  ***Adenomus kelaartii*** | https://www.morphosource.org/concern/media/000024953?locale=en | [ark:/87602/m4/M24953](http://n2t.net/ark:/87602/m4/M24953)  https://doi.org/10.17602/M2/M24953 |
| 95 | CT scan of body CT scan of cas:herp:162120  ***Altiphrynoides malcomi*** | https://www.morphosource.org/concern/media/000024979?locale=en | [ark:/87602/m4/M24979](http://n2t.net/ark:/87602/m4/M24979)  https://doi.org/10.17602/M2/M24979 |
| 96 | Full body STL CT scan of uf:herp:3997  ***Anaxyrus fowleri*** | https://www.morphosource.org/concern/media/000011366?locale=en | [ark:/87602/m4/M11366](http://n2t.net/ark:/87602/m4/M11366)  https://doi.org/10.17602/M2/M11366 |
| 97 | CT scan of uf:herp:124269  ***Anaxyrus quercicus*** | https://www.morphosource.org/concern/media/000011372?locale=en | [ark:/87602/m4/M11372](http://n2t.net/ark:/87602/m4/M11372)  https://doi.org/10.17602/M2/M11372 |
| 98 | CT scan of uf:herp:3170  ***Anaxyrus terrestris*** | https://www.morphosource.org/concern/media/000011374?locale=en | [doi:10.17602/M2/M11374](https://doi.org/10.17602/M2/M11374) |
| 99 | CT scan of body ku:kuh:334742  ***Ansonia mcgregori*** | https://www.morphosource.org/concern/media/000024984?locale=en | [ark:/87602/m4/M24984](http://n2t.net/ark:/87602/m4/M24984)  https://doi.org/10.17602/M2/M24984 |
| 100 | Whole body STL: UF-H-39140 ***Atelopus ignescens*** | https://www.morphosource.org/concern/media/000011496?locale=en | [ark:/87602/m4/M98142](http://n2t.net/ark:/87602/m4/M98142)  <https://doi.org/10.17602/M2/M11496> |
| 101 | Full body STL CT scan of uf:herp:93190  ***Atelopus oxyrhynchus*** | https://www.morphosource.org/concern/media/000011363?locale=en | [ark:/87602/m4/M11363](http://n2t.net/ark:/87602/m4/M11363)  https://doi.org/10.17602/M2/M11363 |
| 102 | CT scan of body of cas:herp:156600  ***Capensibufo sp.*** | https://www.morphosource.org/concern/media/000025498?locale=en | [ark:/87602/m4/M25498](http://n2t.net/ark:/87602/m4/M25498)  https://doi.org/10.17602/M2/M25498 |
| 103 | CT scan of body CT scan of cas:herp:162676  ***Didynamipus sjostedti*** | https://www.morphosource.org/concern/media/000023005?locale=en | [ark:/87602/m4/M23005](http://n2t.net/ark:/87602/m4/M23005)  https://doi.org/10.17602/M2/M23005 |
| 104 | CT scan of body CT scan of cas:herp:227515  ***Duttaphrynus dodsoni*** | https://www.morphosource.org/concern/media/000025585?locale=en | [ark:/87602/m4/M25585](http://n2t.net/ark:/87602/m4/M25585)  https://doi.org/10.17602/M2/M25585 |
| 105 | STL of skeleton CT scan of USNM-565102, ***Frostius pernambucensis*** | https://www.morphosource.org/concern/media/000032526?locale=en | [ark:/87602/m4/M32526](http://n2t.net/ark:/87602/m4/M32526)  https://doi.org/10.17602/M2/M32526 |
| 106 | 3D Full Body: microCT scan of rmca:vert:B.105045  ***Laurentophryne parkeri*** | https://www.morphosource.org/concern/media/000031690?locale=en | [ark:/87602/m4/M31690](http://n2t.net/ark:/87602/m4/M31690)  https://doi.org/10.17602/M2/M31690 |
| 107 | CT scan of body of uf:herp:63183  ***Melanophryniscus stelzneri*** | https://www.morphosource.org/concern/media/000012431?locale=en | [ark:/87602/m4/M12431](http://n2t.net/ark:/87602/m4/M12431)  https://doi.org/10.17602/M2/M12431 |
| 108 | CT scan of body of cas:herp:162553  ***Mertensophryne micranotis*** | https://www.morphosource.org/concern/media/000025588?locale=en | [ark:/87602/m4/M25588](http://n2t.net/ark:/87602/m4/M25588)  https://doi.org/10.17602/M2/M25588 |
| 109 | CT scan of body of cas:herp:258098  ***Nectophryne afra*** | https://www.morphosource.org/concern/media/000026320?locale=en | [ark:/87602/m4/M26320](http://n2t.net/ark:/87602/m4/M26320)  https://doi.org/10.17602/M2/M26320 |
| 110 | CT scan of body of USNM: Amphibians & Reptiles:217402  ***Nectophrynoides viviparus*** | https://www.morphosource.org/concern/media/000381423?locale=en | [ark:/87602/m4/381423](http://n2t.net/ark:/87602/m4/381423)  https://doi.org/10.17602/M2/M381423 |
| 111 | CT scan of skeleton ku:kuh:310635  ***Pelophryne lighti*** | https://www.morphosource.org/concern/media/000031579?locale=en | [ark:/87602/m4/M31579](http://n2t.net/ark:/87602/m4/M31579)  https://doi.org/10.17602/M2/M31579 |
| 112 | CT scan of of uf:herp:104862  ***Peltophryne guentheri*** | https://www.morphosource.org/concern/media/000011379?locale=en | [ark:/87602/m4/M11379](http://n2t.net/ark:/87602/m4/M11379)  https://doi.org/10.17602/M2/M11379 |
| 113 | STL of body CT scan of KU-96839, ***Truebella skoptes*** | https://www.morphosource.org/concern/media/000039601?locale=en | [ark:/87602/m4/M39601](http://n2t.net/ark:/87602/m4/M39601)  https://doi.org/10.17602/M2/M39601 |
| 114 | STL of body CT scan of KU-kuh-196599, ***Truebella tothastes*** | https://www.morphosource.org/concern/media/000039599?locale=en | [ark:/87602/m4/M39599](http://n2t.net/ark:/87602/m4/M39599)  https://doi.org/10.17602/M2/M39599 |
| 115 | STL of skeleton CT scan of KU-H-166716, ***Allophryne ruthveni*** | https://www.morphosource.org/concern/media/000026338?locale=en | [ark:/87602/m4/M26338](http://n2t.net/ark:/87602/m4/M26338)  https://doi.org/10.17602/M2/M26338 |
| 116 | CT scan of body of uf:herp:30578  ***Centrolene buckleyi*** | https://www.morphosource.org/concern/media/000016296?locale=en | [ark:/87602/m4/M16296](http://n2t.net/ark:/87602/m4/M16296)  https://doi.org/10.17602/M2/M16296 |
| 117 | CT scan of body of uf:herp:30579  ***Centrolene buckleyi*** | https://www.morphosource.org/concern/media/000012442?locale=en | [ark:/87602/m4/M12442](http://n2t.net/ark:/87602/m4/M12442)  https://doi.org/10.17602/M2/M12442 |
| 118 | CT scan of body of uf:herp:137267  ***Cochranella granulosa*** | https://www.morphosource.org/concern/media/000016298?locale=en | [ark:/87602/m4/M16298](http://n2t.net/ark:/87602/m4/M16298)  https://doi.org/10.17602/M2/M16298 |
| 119 | CT scan of body of uf:herp:140640  ***Hyalinobatrachium fleischmanni*** | https://www.morphosource.org/concern/media/000016300?locale=en | [ark:/87602/m4/M16300](http://n2t.net/ark:/87602/m4/M16300)  https://doi.org/10.17602/M2/M16300 |
| 120 | STL of skeleton cm:herps:68338  ***Cycloramphus asper*** | https://www.morphosource.org/concern/media/000022882?locale=en | [ark:/87602/m4/M22882](http://n2t.net/ark:/87602/m4/M22882)  https://doi.org/10.17602/M2/M22882 |
| 121 | CT scan of body of cm:herps:63926  ***Eupsophus roseus*** | https://www.morphosource.org/concern/media/000021082?locale=en | [ark:/87602/m4/M21082](http://n2t.net/ark:/87602/m4/M21082)  https://doi.org/10.17602/M2/M21082 |
| 122 | CT scan of body of cas:herp:141996  ***Hylorina sylvatica*** | https://www.morphosource.org/concern/media/000022182?locale=en | [ark:/87602/m4/M22182](http://n2t.net/ark:/87602/m4/M22182)  https://doi.org/10.17602/M2/M22182 |
| 123 | STL of Skeleton CT scan of USNM200455, ***Macrogenioglottus alipioi*** | https://www.morphosource.org/concern/media/000036028?locale=en | [ark:/87602/m4/M36028](http://n2t.net/ark:/87602/m4/M36028)  https://doi.org/10.17602/M2/M36028 |
| 124 | CT scan of body CT scan of cm:herps:68357  ***Thoropa miliaris*** | https://www.morphosource.org/concern/media/000024444?locale=en | [ark:/87602/m4/M24444](http://n2t.net/ark:/87602/m4/M24444)  https://doi.org/10.17602/M2/M24444 |
| 125 | CT scan of body cas:sua:11907  ***Zachaenus parvulus*** | https://www.morphosource.org/concern/media/000023008?locale=en | [ark:/87602/m4/M23008](http://n2t.net/ark:/87602/m4/M23008)  https://doi.org/10.17602/M2/M23008 |
| 126 | CT scan of ces:f:203  ***Nasikabatrachus sahyadrensis*** | https://www.morphosource.org/concern/media/000019813?locale=en | [ark:/87602/m4/M19813](http://n2t.net/ark:/87602/m4/M19813)  https://doi.org/10.17602/M2/M19813 |
| 127 | STL of skeleton CT scan of cas:herp:156991  ***Seychellophryne gardineri*** (*Sooglossus gardineri*) | https://www.morphosource.org/concern/media/000025831?locale=en | [ark:/87602/m4/M25831](http://n2t.net/ark:/87602/m4/M25831)  https://doi.org/10.17602/M2/M25831 |
| 128 | STL of skeleton CT scan of CAS-H-160084, ***Sooglossus sechellensis*** | https://www.morphosource.org/concern/media/000025840?locale=en | [ark:/87602/m4/M25840](http://n2t.net/ark:/87602/m4/M25840)  https://doi.org/10.17602/M2/M25840 |
| 129 | STL of skeleton CT scan of uf:herp:68008  ***Syncope antenori*** | https://www.morphosource.org/concern/media/000016850?locale=en | [ark:/87602/m4/M16850](http://n2t.net/ark:/87602/m4/M16850)  https://doi.org/10.17602/M2/M16850 |
| 130 | STL of skeleton CT scan of ces:f:1807  ***Melanobatrachus indicus*** | https://www.morphosource.org/concern/media/000019810?locale=en | [ark:/87602/m4/M19810](http://n2t.net/ark:/87602/m4/M19810)  https://doi.org/10.17602/M2/M19810 |
| 131 | STL of skeleton uf:herp:9034  ***Chiasmocleis crucis*** | https://www.morphosource.org/concern/media/000024429?locale=en | [ark:/87602/m4/M24429](http://n2t.net/ark:/87602/m4/M24429)  https://doi.org/10.17602/M2/M24429 |
| 132 | Mesh skeleton of ummz:herps:219339  ***Cophyla phyllodactyla*** | https://www.morphosource.org/concern/media/000045194?locale=en | [ark:/87602/m4/M45194](http://n2t.net/ark:/87602/m4/M45194)  https://doi.org/10.17602/M2/M45194 |
| 133 | STL of skeleton CT scan of uf:herp:110645  ***Gastrophryne carolinensis*** | https://www.morphosource.org/concern/media/000020081?locale=en | [doi:10.17602/M2/M20081](https://doi.org/10.17602/M2/M20081) |
| 134 | MicroCT volume and derivatives of amnh:herpetology:a-131554, ***Otophryne robusta*** | https://www.morphosource.org/concern/media/000070892?locale=en | [ark:/87602/m4/M70892](http://n2t.net/ark:/87602/m4/M70892)  https://doi.org/10.17602/M2/M70892 |
| 135 | Mesh file (STL) microCT volume and derivatives lsumz:herps:95004  ***Paedophryne amauensis*** | https://www.morphosource.org/concern/media/000075318?locale=en | [ark:/87602/m4/M75318](http://n2t.net/ark:/87602/m4/M75318)  https://doi.org/10.17602/M2/M75318 |
| 136 | STL of skeleton microCT volume and derivatives mcz:herp:a-13818 ***Parhoplophryne usambaricus*** | https://www.morphosource.org/concern/media/000042754?locale=en | [ark:/87602/m4/M42754](http://n2t.net/ark:/87602/m4/M42754)  https://doi.org/10.17602/M2/M42754 |
| 137 | STL of skeleton uf:herp:15363  ***Stumpffia pygmaea*** | https://www.morphosource.org/concern/media/000024425?locale=en | [ark:/87602/m4/M24425](http://n2t.net/ark:/87602/m4/M24425)  https://doi.org/10.17602/M2/M24425 |
| 138 | MicroCT volume and derivatives of amnh:herpetology:a-90935, ***Synapturanus mirandaribeiroi*** | https://www.morphosource.org/concern/media/000070894?locale=en | [ark:/87602/m4/M70894](http://n2t.net/ark:/87602/m4/M70894)  https://doi.org/10.17602/M2/M70894 |
| 139 | STL of skeleton uf:herp:20016  ***Uperodon montanus*** | https://www.morphosource.org/concern/media/000024427?locale=en | [ark:/87602/m4/M24427](http://n2t.net/ark:/87602/m4/M24427)  https://doi.org/10.17602/M2/M24427 |
| 140 | STL of skeleton CT scan of CAS-H-85287, ***Uperodon systoma*** | https://www.morphosource.org/concern/media/000026358?locale=en | [ark:/87602/m4/M26358](http://n2t.net/ark:/87602/m4/M26358)  https://doi.org/10.17602/M2/M26358 |
| 141 | STL of skeleton CT scan of cas:herp:258533  ***Hemisus guineensis*** | https://www.morphosource.org/concern/media/000015807?locale=en | [ark:/87602/m4/M15807](http://n2t.net/ark:/87602/m4/M15807)  https://doi.org/10.17602/M2/M15807 |
| 142 | CT scan of body of cas:herp:167436  ***Balebreviceps hillmani*** | https://www.morphosource.org/concern/media/000022198?locale=en | [ark:/87602/m4/M22198](http://n2t.net/ark:/87602/m4/M22198)  https://doi.org/10.17602/M2/M22198 |
| 143 | CT scan of body of cas:herp:168818  ***Callulina kisiwamsitu*** | https://www.morphosource.org/concern/media/000022207?locale=en | [ark:/87602/m4/M22207](http://n2t.net/ark:/87602/m4/M22207)  https://doi.org/10.17602/M2/M22207 |
| 144 | STL of skeleton CT scan of uf:herp:111075  ***Probreviceps macrodactylus*** | https://www.morphosource.org/concern/media/000020038?locale=en | [ark:/87602/m4/M20038](http://n2t.net/ark:/87602/m4/M20038)  https://doi.org/10.17602/M2/M20038 |
| 145 | CT scan of body of cas:herp:246447  ***Probreviceps macrodactylus*** | https://www.morphosource.org/concern/media/000024956?locale=en | [ark:/87602/m4/M24956](http://n2t.net/ark:/87602/m4/M24956)  https://doi.org/10.17602/M2/M24956 |
| 146 | CT scan of body of CAS-H-11521, ***Spelaeophryne methneri*** | https://www.morphosource.org/concern/media/000024970?locale=en | [ark:/87602/m4/M24970](http://n2t.net/ark:/87602/m4/M24970)  https://doi.org/10.17602/M2/M24970 |
| 147 | CT scan of body cas:herp:207285  ***Arthroleptis bioko*** | <https://www.morphosource.org/concern/biological_specimens/0000S8516>  SPECIMEN LINK NOT MEDIA | [ark:/87602/m4/M21085](http://n2t.net/ark:/87602/m4/M21085)  https://doi.org/10.17602/M2/M21085 |
| 148 | CT scan of body cas:herp:254126  ***Astylosternus diadematus*** | https://www.morphosource.org/concern/media/000021509?locale=en | [ark:/87602/m4/M21509](http://n2t.net/ark:/87602/m4/M21509)  https://doi.org/10.17602/M2/M21509 |
| 149 | CT scan of body cas:herp:250950  ***Cardioglossa cyaneospila*** | https://www.morphosource.org/concern/media/000021506?locale=en | [ark:/87602/m4/M21506](http://n2t.net/ark:/87602/m4/M21506)  https://doi.org/10.17602/M2/M21506 |
| 150 | CT scan of body cas:herp:253933  ***Leptodactylodon ovatus*** | https://www.morphosource.org/concern/media/000021512?locale=en | [ark:/87602/m4/M21512](http://n2t.net/ark:/87602/m4/M21512)  https://doi.org/10.17602/M2/M21512 |
| 151 | Mesh file (STL) CT scan of CAS260137, ***Leptopelis brevirostris*** | https://www.morphosource.org/concern/media/000070544?locale=en | [ark:/87602/m4/M70544](http://n2t.net/ark:/87602/m4/M70544)  https://doi.org/10.17602/M2/M70544 |
| 152 | CT scan of body cas:herp:253554  ***Leptopelis notatus*** | https://www.morphosource.org/concern/media/000021518?locale=en | [ark:/87602/m4/M21518](http://n2t.net/ark:/87602/m4/M21518)  https://doi.org/10.17602/M2/M21518 |
| 153 | CT scan of body cas:herp:256829  ***Nyctibates corrugatus*** | https://www.morphosource.org/concern/media/000021523?locale=en | [ark:/87602/m4/M21523](http://n2t.net/ark:/87602/m4/M21523)  https://doi.org/10.17602/M2/M21523 |
| 154 | STL of skeleton CT scan of cas-herp-254134, ***Trichobatrachus robustus*** | https://www.morphosource.org/concern/media/000027164?locale=en | [ark:/87602/m4/M27164](http://n2t.net/ark:/87602/m4/M27164)  https://doi.org/10.17602/M2/M27164 |
| 155 | STL of Skeleton CT scan of CAS-H-168486, ***Phrynobatrachus krefftii*** | https://www.morphosource.org/concern/media/000025444?locale=en | [ark:/87602/m4/M25444](http://n2t.net/ark:/87602/m4/M25444)  https://doi.org/10.17602/M2/M25444 |
| 156 | STL of skeleton CT scan of cas:herp:230204  ***Odontobatrachus natator*** | https://www.morphosource.org/concern/media/000024128?locale=en | [ark:/87602/m4/M24128](http://n2t.net/ark:/87602/m4/M24128)  https://doi.org/10.17602/M2/M24128 |
| 157 | STL of skeleton CT scan of CAS-H-154657, ***Hildebrandtia ornata*** | https://www.morphosource.org/concern/media/000024510?locale=en | [ark:/87602/m4/M24510](http://n2t.net/ark:/87602/m4/M24510)  https://doi.org/10.17602/M2/M24510 |
| 158 | CT scan of skeleton of cas-herp-256862, ***Ptychadena oxyrhynchus*** | https://www.morphosource.org/concern/media/000040065?locale=en | [ark:/87602/m4/M40065](http://n2t.net/ark:/87602/m4/M40065)  https://doi.org/10.17602/M2/M40065 |
| 159 | STL of skeleton CT scan of CAS-H-156428, ***Anhydrophryne rattrayi*** | https://www.morphosource.org/concern/media/000024514?locale=en | [ark:/87602/m4/M24514](http://n2t.net/ark:/87602/m4/M24514)  https://doi.org/10.17602/M2/M24514 |
| 160 | STL of Skeleton CT scan of CAS-H-157023, ***Arthroleptella lightfooti*** | https://www.morphosource.org/concern/media/000025077?locale=en | [ark:/87602/m4/M25077](http://n2t.net/ark:/87602/m4/M25077)  https://doi.org/10.17602/M2/M25077 |
| 161 | STL of Skeleton CT scan of CAS-H-125890, ***Cacosternum boettgeri*** | https://www.morphosource.org/concern/media/000024502?locale=en | [ark:/87602/m4/M24502](http://n2t.net/ark:/87602/m4/M24502)  https://doi.org/10.17602/M2/M24502 |
| 162 | STL of Skeleton CT scan of CAS-H-156975, ***Cacosternum namaquense*** | https://www.morphosource.org/concern/media/000025450?locale=en | [ark:/87602/m4/M25450](http://n2t.net/ark:/87602/m4/M25450)  https://doi.org/10.17602/M2/M25450 |
| 163 | STL of Skeleton CT scan of CAS-H-157008, ***Mitrobatrachella capensis*** | https://www.morphosource.org/concern/media/000025575?locale=en | [ark:/87602/m4/M25575](http://n2t.net/ark:/87602/m4/M25575)  https://doi.org/10.17602/M2/M25575 |
| 164 | STL of skeleton CT scan of CAS-H-156617, ***Natalobatrachus bonebergi*** | https://www.morphosource.org/concern/media/000024517?locale=en | [ark:/87602/m4/M24517](http://n2t.net/ark:/87602/m4/M24517)  https://doi.org/10.17602/M2/M24517 |
| 165 | STL of Skeleton CT scan of CAS-H-156126, ***Nothophryne broadley*** | https://www.morphosource.org/concern/media/000025577?locale=en | [ark:/87602/m4/M25577](http://n2t.net/ark:/87602/m4/M25577)  https://doi.org/10.17602/M2/M25577 |
| 166 | STL of skeleton CT scan of CAS-H-165936, ***Poyntonia paludicola*** | https://www.morphosource.org/concern/media/000025584?locale=en | [ark:/87602/m4/M25584](http://n2t.net/ark:/87602/m4/M25584)  https://doi.org/10.17602/M2/M25584 |
| 167 | STL of Skeleton CT scan of UF-H-92093, ***Pyxicephalus adspersus*** | https://www.morphosource.org/concern/media/000025343?locale=en | [ark:/87602/m4/M25343](http://n2t.net/ark:/87602/m4/M25343)  https://doi.org/10.17602/M2/M25343 |
| 168 | STL of skeleton CT scan of CAS-H-157511, ***Tomopterna delandii*** | https://www.morphosource.org/concern/media/000025594?locale=en | [ark:/87602/m4/M25594](http://n2t.net/ark:/87602/m4/M25594)  https://doi.org/10.17602/M2/M25594 |
| 169 | CT scan of CAS-H-183290, ***Tomopterna marmorata*** | https://www.morphosource.org/concern/media/000025597?locale=en | [ark:/87602/m4/M25597](http://n2t.net/ark:/87602/m4/M25597)  https://doi.org/10.17602/M2/M25597 |
| 170 | STL of Skeleton CT scan of CAS-H-127500, ***Tomopterna tuberculosa*** | https://www.morphosource.org/concern/media/000025063?locale=en | [ark:/87602/m4/M25063](http://n2t.net/ark:/87602/m4/M25063)  https://doi.org/10.17602/M2/M25063 |
| 171 | STL of skeleton CT scan of UF-H-64720, ***Conraua goliath*** | https://www.morphosource.org/concern/media/000039477?locale=en | [doi:10.17602/M2/M39477](https://doi.org/10.17602/M2/M39477) |
| 172 | CT scan of body cas:herp:258122  ***Conraua crassipes*** | https://www.morphosource.org/concern/media/000022660?locale=en | [ark:/87602/m4/M22660](http://n2t.net/ark:/87602/m4/M22660)  https://doi.org/10.17602/M2/M22660 |
| 173 | STL of skeleton CT scan of CAS-H-260140, ***Petropedetes sp*** | https://www.morphosource.org/concern/media/000025403?locale=en | [ark:/87602/m4/M25403](http://n2t.net/ark:/87602/m4/M25403)  https://doi.org/10.17602/M2/M25403 |
| 174 | STL of skeleton CT scan of CAS-H-103349, ***Petropedetes cf. vulpiae*** | https://www.morphosource.org/concern/media/000025032?locale=en | [ark:/87602/m4/M25032](http://n2t.net/ark:/87602/m4/M25032)  https://doi.org/10.17602/M2/M25032 |
| 175 | STL of Skeleton CT scan of CAS-H-153715, ***Petropedetes palmipes*** | https://www.morphosource.org/concern/media/000025447?locale=en | [ark:/87602/m4/M25447](http://n2t.net/ark:/87602/m4/M25447)  https://doi.org/10.17602/M2/M25447 |
| 176 | STL of body CT scan of UF80585, ***Ceratobatrachus guentheri*** | https://www.morphosource.org/concern/media/000037444?locale=en | [ark:/87602/m4/M37444](http://n2t.net/ark:/87602/m4/M37444)  https://doi.org/10.17602/M2/M37444 |
| 177 | CT scan of body ku:kuh:341253  ***Platymantis guppyi***  (*Cornufer guppyi*) | https://www.morphosource.org/concern/media/000022659?locale=en | [ark:/87602/m4/M22659](http://n2t.net/ark:/87602/m4/M22659)  https://doi.org/10.17602/M2/M22659 |
| 178 | CT scan of body ku:kuh:313722  ***Platymantis polillensis*** | https://www.morphosource.org/concern/media/000024435?locale=en | [ark:/87602/m4/M24435](http://n2t.net/ark:/87602/m4/M24435)  https://doi.org/10.17602/M2/M24435 |
| 179 | STL of skeleton CT scan of ***Fejervarya limnocharis*** CAS-H-23509 | https://www.morphosource.org/concern/media/000024025?locale=en | [ark:/87602/m4/M24025](http://n2t.net/ark:/87602/m4/M24025)  https://doi.org/10.17602/M2/M24025 |
| 180 | STL of body CT scan of CAS258174, ***Hoplobatrachus occipitalis*** | https://www.morphosource.org/concern/media/000037616?locale=en | [ark:/87602/m4/M37616](http://n2t.net/ark:/87602/m4/M37616)  https://doi.org/10.17602/M2/M37616 |
| 181 | STL of skeleton CT scan cas:herp:240435  ***Limnonectes blythii*** | https://www.morphosource.org/concern/media/000024451?locale=en | [ark:/87602/m4/M24451](http://n2t.net/ark:/87602/m4/M24451)  https://doi.org/10.17602/M2/M24451 |
| 182 | CT scan of body ku:kuh:306056  ***Limnonectes macrocephalus*** | https://www.morphosource.org/concern/media/000020287?locale=en | [ark:/87602/m4/M20287](http://n2t.net/ark:/87602/m4/M20287)  https://doi.org/10.17602/M2/M20287 |
| 183 | Skeleton STL CT scan of ku:kuh:320174  ***Occidozyga laevis*** | https://www.morphosource.org/concern/media/000033050?locale=en | [ark:/87602/m4/M33050](http://n2t.net/ark:/87602/m4/M33050)  https://doi.org/10.17602/M2/M33050 |
| 184 | Mesh file (STL) CT scan of fmnh-amphibians and reptiles-81229, ***Lankanectes corrugatus*** | https://www.morphosource.org/concern/media/000058041?locale=en | [ark:/87602/m4/M58041](http://n2t.net/ark:/87602/m4/M58041)  https://doi.org/10.17602/M2/M58041 |
| 185 | STL of skeleton CT scan of ces:f:775  ***Nyctibatrachus aliciae*** | https://www.morphosource.org/concern/media/000019815?locale=en | [ark:/87602/m4/M19815](http://n2t.net/ark:/87602/m4/M19815)  https://doi.org/10.17602/M2/M19815 |
| 186 | STL of Skeleton CT scan of ***Nyctibatrachus major*** CAS-H-125402 | https://www.morphosource.org/concern/media/000024179?locale=en | [ark:/87602/m4/M24179](http://n2t.net/ark:/87602/m4/M24179)  https://doi.org/10.17602/M2/M24179 |
| 187 | STL of skeleton CT scan of cas:herp:258101  ***Hylarana albolabris*** | https://www.morphosource.org/concern/media/000020034?locale=en | [ark:/87602/m4/M20034](http://n2t.net/ark:/87602/m4/M20034)  https://doi.org/10.17602/M2/M20034 |
| 188 | MicroCT volume and derivatives uf:herp:87142  ***Rana capito*** | https://www.morphosource.org/concern/media/000065728?locale=en | [ark:/87602/m4/M65728](http://n2t.net/ark:/87602/m4/M65728)  https://doi.org/10.17602/M2/M65728 |
| 189 | microCT volume and derivatives uf:herp:24687  ***Rana capito*** | https://www.morphosource.org/concern/media/000065723?locale=en | [ark:/87602/m4/M65723](http://n2t.net/ark:/87602/m4/M65723)  https://doi.org/10.17602/M2/M65723 |
| 190 | Mesh file (STL) microCT volume and derivatives uf:herp:163449  ***Rana capito*** | https://www.morphosource.org/concern/media/000064161?locale=en | [ark:/87602/m4/M64161](http://n2t.net/ark:/87602/m4/M64161) https://doi.org/10.17602/M2/M64161 |
| 191 | Mesh file (STL) microCT volume and derivatives uf:herp:159097  ***Rana capito*** | https://www.morphosource.org/concern/media/000064160?locale=en | [ark:/87602/m4/M64160](http://n2t.net/ark:/87602/m4/M64160) https://doi.org/10.17602/M2/M64160 |
| 192 | Mesh file (STL) microCT volume and derivatives uf:herp:103333 ***Rana capito*** | https://www.morphosource.org/concern/media/000064163?locale=en | [ark:/87602/m4/M64163](http://n2t.net/ark:/87602/m4/M64163)  https://doi.org/10.17602/M2/M64163 |
| 193 | Mesh file (STL) microCT volume and derivatives uf:herp:103332  ***Rana capito*** | https://www.morphosource.org/concern/media/000064162?locale=en | [ark:/87602/m4/M64162](http://n2t.net/ark:/87602/m4/M64162)  https://doi.org/10.17602/M2/M64162 |
| 194 | microCT volume and derivatives NCSM:Herp:9118  ***Rana sp.*** | https://www.morphosource.org/concern/media/000085478?locale=en | [ark:/87602/m4/M85478](http://n2t.net/ark:/87602/m4/M85478)  https://doi.org/10.17602/M2/M85478 |
| 195 | STL of skeleton CT scan of uf:herp:76511  ***Lithobates clamitans*** | https://www.morphosource.org/concern/media/000020064?locale=en | [doi:10.17602/M2/M20064](https://doi.org/10.17602/M2/M20064) |
| 196 | Mesh file (STL) microCT volume and derivatives cas:herp:104235  ***Rana leptodactyla*** | https://www.morphosource.org/concern/media/000062532?locale=en | [ark:/87602/m4/M62532](http://n2t.net/ark:/87602/m4/M62532)  https://doi.org/10.17602/M2/M62532 |
| 197 | Mesh ummz:herps:240539  ***Rana tigerina*** | https://www.morphosource.org/concern/media/000057187?locale=en | [ark:/87602/m4/M57187](http://n2t.net/ark:/87602/m4/M57187)  https://doi.org/10.17602/M2/M57187 |
| 198 | STL of skeleton CT scan of ***Micrixalus cf adonis*** CESF1647 | https://www.morphosource.org/concern/media/000019816?locale=en | [ark:/87602/m4/M19816](http://n2t.net/ark:/87602/m4/M19816)  https://doi.org/10.17602/M2/M19816 |
| 199 | STL of skeleton CT scan of ***Micrixalus herre*** CAS SUA 7265 | https://www.morphosource.org/concern/media/000024356?locale=en | [ark:/87602/m4/M24356](http://n2t.net/ark:/87602/m4/M24356)  https://doi.org/10.17602/M2/M24356 |
| 200 | STL of skeleton CT scan of ces:f:648  ***Indirana phrynoderma*** | https://www.morphosource.org/concern/media/000019819?locale=en | [ark:/87602/m4/M19819](http://n2t.net/ark:/87602/m4/M19819)  https://doi.org/10.17602/M2/M19819 |
| 201 | CT scan of body CT scan of cas:herp:250237  ***Boophis boehmei*** | https://www.morphosource.org/concern/media/000024352?locale=en | [ark:/87602/m4/M24352](http://n2t.net/ark:/87602/m4/M24352)  https://doi.org/10.17602/M2/M24352 |
| 202 | CT scan of CAS-H-211461, ***Buergeria japonica*** | https://www.morphosource.org/concern/media/000025602?locale=en | [ark:/87602/m4/M25602](http://n2t.net/ark:/87602/m4/M25602)  https://doi.org/10.17602/M2/M25602 |
| 203 | STL of Skeleton CT scan of CAS-H-253330, ***Chiromantis rufescens*** | https://www.morphosource.org/concern/media/000025659?locale=en | [ark:/87602/m4/M25659](http://n2t.net/ark:/87602/m4/M25659)  https://doi.org/10.17602/M2/M25659 |
| 204 | CT scan of CAS-H-247492, ***Nyctixalus pictus*** | https://www.morphosource.org/concern/media/000025668?locale=en | [ark:/87602/m4/M25668](http://n2t.net/ark:/87602/m4/M25668)  https://doi.org/10.17602/M2/M25668 |
| 205 | STL of Skeleton CT scan of KU-H-321404, ***Nyctixalus spinosus*** | https://www.morphosource.org/concern/media/000025665?locale=en | [ark:/87602/m4/M25665](http://n2t.net/ark:/87602/m4/M25665)  https://doi.org/10.17602/M2/M25665 |
| 206 | STL of Skeleton CT scan of CAS-H-SU20916, ***Nyctixalus spinosus*** | https://www.morphosource.org/concern/media/000025662?locale=en | [ark:/87602/m4/M25662](http://n2t.net/ark:/87602/m4/M25662)  https://doi.org/10.17602/M2/M25662 |
| 207 | STL of skeleton CT scan of KU-H-321696, ***Philautus surdus*** | https://www.morphosource.org/concern/media/000025813?locale=en | [ark:/87602/m4/M25813](http://n2t.net/ark:/87602/m4/M25813)  https://doi.org/10.17602/M2/M25813 |
| 208 | CT scan of KU-H-335721, ***Philautus petersi*** | https://www.morphosource.org/concern/media/000025672?locale=en | [ark:/87602/m4/M25672](http://n2t.net/ark:/87602/m4/M25672)  https://doi.org/10.17602/M2/M25672 |
| 209 | CT scan of KU-H-335710, ***Philautus petersi*** | https://www.morphosource.org/concern/media/000025670?locale=en | [ark:/87602/m4/M25670](http://n2t.net/ark:/87602/m4/M25670)  https://doi.org/10.17602/M2/M25670 |
| 210 | STL of skeleton CT scan of KU-H-321598, ***Philautus worcesteri*** | https://www.morphosource.org/concern/media/000025843?locale=en | [ark:/87602/m4/M25843](http://n2t.net/ark:/87602/m4/M25843)  https://doi.org/10.17602/M2/M25843 |
| 211 | CT scan of KU-H-327008, ***Polypedates macrotis*** | https://www.morphosource.org/concern/media/000025848?locale=en | [ark:/87602/m4/M25848](http://n2t.net/ark:/87602/m4/M25848)  https://doi.org/10.17602/M2/M25848 |
| 212 | CT scan of KU-H-311857, ***Polypedates nigripunctatus*** | https://www.morphosource.org/concern/media/000025854?locale=en | [ark:/87602/m4/M25854](http://n2t.net/ark:/87602/m4/M25854)  https://doi.org/10.17602/M2/M25854 |
| 213 | STL of skeleton CT scan of ***Raorchestes chalazodes*** CESF-2121 | https://www.morphosource.org/concern/media/000019820?locale=en | [ark:/87602/m4/M19820](http://n2t.net/ark:/87602/m4/M19820)  https://doi.org/10.17602/M2/M19820 |
| 214 | STL of skeleton CT scan of ***Raorchestes flaviocularas*** CES-F-1253 | https://www.morphosource.org/concern/media/000019827?locale=en | [ark:/87602/m4/M19827](http://n2t.net/ark:/87602/m4/M19827)  https://doi.org/10.17602/M2/M19827 |
| 215 | STL of skeleton CT scan of ***Raorchestes manohari*** CESF2121 | https://www.morphosource.org/concern/media/000019817?locale=en | [ark:/87602/m4/M19817](http://n2t.net/ark:/87602/m4/M19817)  https://doi.org/10.17602/M2/M19817 |
| 216 | STL of skeleton CT scan of ***Raorchestes nerostegona*** CESF1269 | https://www.morphosource.org/concern/media/000019831?locale=en | [ark:/87602/m4/M19831](http://n2t.net/ark:/87602/m4/M19831)  https://doi.org/10.17602/M2/M19831 |
| 217 | STL of skeleton CT scan of ***Raorchestes ochlandrae*** CESF2185 | https://www.morphosource.org/concern/media/000019824?locale=en | [ark:/87602/m4/M19824](http://n2t.net/ark:/87602/m4/M19824)  https://doi.org/10.17602/M2/M19824 |
| 218 | STL of skeleton CT scan of ces:f:1283  ***Raorchestes primarrumpfi*** | https://www.morphosource.org/concern/media/000019821?locale=en | [ark:/87602/m4/M19821](http://n2t.net/ark:/87602/m4/M19821)  https://doi.org/10.17602/M2/M19821 |
| 219 | STL of skeleton CT scan of ***Raorchestes resplendens*** CESF1325 | https://www.morphosource.org/concern/media/000019826?locale=en | [ark:/87602/m4/M19826](http://n2t.net/ark:/87602/m4/M19826)  https://doi.org/10.17602/M2/M19826 |

1. [ark:/87602/m4/M98142](http://n2t.net/ark:/87602/m4/M98142) THIS IS A CT SCAN NO PIXEL SPACING
