# Supplementary material for: An overview of the osseous palmar sesamoid in Anura, with the particular case of some Rhinella species
Source: PeerJ. 2023 May 15;11:e15063. doi: 10.7717/peerj.15063 (PMC10194070; doi:10.7717/peerj.15063)
Supplement: Supplemental Information 2 — Comparative table of the palmar sesamoid and associated anatomical systems in 4 species of Rhinella genus. [file peerj-11-15063-s002.pdf]

Structure	*R. arenarum*	*R. dorbigny*	*R. margaritifera*	*R. diptycha*
Palmar Aponeurosis	Absent	Absent	Absent	It is a thin layer that covers all the surface of the ventral hand muscles, reaching the basal phalanx. Over the carpals it is thicker than in the other hand regions.
Flexor digitorumcom munis (Fig. 1c, e)	It is a strong muscle, fusiform that lies along the radio-ulna. It has two origins, one is long, wide tendinous from the distal condyle of the humerus; and the other, is a short, narrow, and tubular tendon coming from the medial condyle of the humerus. Both origins are contiguous with each other.	The muscle is fusiform and thick that inserts on the flexor plate by a short, wide, and thick tendon. It lays superficial on the ventral surface of the antebrachium. It has three branches. Two superficial branches well defined origin from the medial epicondyle of the humerus. The lateral one arises by a thick, ribbon-like tendon, longer than the medial branch; and the medial branch by a short and	Fusiform and thin muscle that arises fleshy from the medial epicondyle of the humerus and tendinous from the elbow aponeurosis. It is inserted on the Flexor plate by a thick fan- shaped and long tendon. On the proximal half it is united by some fibers with the m. flexor carpi ulnaris and m. flexor carpi radialis.	Fusiform and thick muscle that arises from the medial epicondyle of the humerus by a ribbon-like tendon. Its distal tendon forms the flexor plate that originatesthe flexor tendonand covers the palmar surface.<br>Some fibers insert on the dorsal surface of the sesamoid.

	The muscle is inserted on the flexor plate by a wide, long, and rectangular tendon. United by fibers to the m. epitrochleo-anconeus, on its deeper face.	thin tendon. The third one is a deep, thin and short branch, with a fleshy origin from the elbow aponeurosis.		
Flexor plate (Fig. 1b, d, f)	A big and rounded plate covers almost all the ventral face of the hand. Tendons Flexors III, IV and V originate from the flexor plate, and inserts on the distal phalanx of the correspondent digits. The tendon of origin of the m. lumbricalis longus IV and V also origins from the flexor plate	A wide flexor plate covers almost all the palmar face of the hand,<br>Flexor tendons III, IV and V originate from the medial and distal edge of the flexor plate, also the tendon of origin of the m. lumbricalis longus III, IV and V.	It arises from the distal tendon of the m. flexor digitorum communis, located centrally on the ventral face of the hand. Tendons flexors II-V arise very close to each other from the distal border of the Flexor plate. They insert on the base of the distal phalanx of each correspondent digit.	A big and rounded plate covers almost all the ventral face of the hand, and continuous with the flexor tendons III, IV and V inserting on the base of the last phalanx of the correspondent digit.
Palmar sesamoid	It is approximately rectangular with a	It is a big, flat (not deep), and round sesamoid embedded in the	An ovoid and big sesamoid is totally embedded forming	It is a big, flat (not deep), and round sesamoid embedded in

(Fig. 1a, c, e)	proximal triangular process. The body of the sesamoid convexity that fits softly into the concavity of the distal carpal III-V bone, constituting a saddle joint. The sesamoid is located on the postaxial axis of the hand.	flexor plate, covering almost all its surface. It is supported by a softly curved ventral surface of the carpal an III-V.	the flexor plate, and lies parallel to the distal tendon of the m. flexor digitorum communis and to the flexor tendons. The sesamoid has a convex surface that articulates with the concave deep depression of the carpal distal III-V, like a hinge joint.	the flexor plate, covering almost all its surface. It is supported by a softly curved ventral surface of the carpal III-V.
Flexor accessorius (Palmaris profundus sensu Gaupp,1896) (Fig. 1a, c)	Square and wide muscle that originates fleshy on the lateral-distal condyle of the ulna. It inserts fleshy on the flexor plate. It extends obliquely on the lateral edge of the flexor plate.	Square short and thick muscle that lies on the proximal and lateral border of the hand. It arises from the lateral condyle of the radio-ulna and inserts on the lateral border of the flexor plate.	It is a rounded muscle that extends obliquely on the ulnar and originates from it and from the lateral extreme of the radio-ulna condyle. It inserts fleshy and in a wide form on the proximal-lateral border of the flexor plate.	It is a short and thick muscle. It originates from the ulnar distal epicondyle and from the lateral surface of the ulnar. It is a fun-shaped muscle that inserts onto the palmar sesamoid proximal extreme.

SM2: Comparative table of the palmar sesamoid and associated anatomical systems in 4 species of *Rhinella* genus.
