# Supplementary material for: An overview of the osseous palmar sesamoid in Anura, with the particular case of some Rhinella species
Source: PeerJ. 2023 May 15;11:e15063. doi: 10.7717/peerj.15063 (PMC10194070; doi:10.7717/peerj.15063)
Supplement: Supplemental Information 3 — Character matrix used in Mesquite 2.7 (Maddison & Maddison, 2019) through character mapping investigate the relationship between the palmar sesamoid, the carpal ventral surface, phylogeny, and the habitat use within the anuran group [file peerj-11-15063-s003.pdf]

TAXA	Palmar sesamoid: O=absent; 1=present	Level of the carpals palmar surface: 0=concave; 1=protruded; 2= flat	Main habitat use: 0=terrestrial; 1= aquatic; 2=arboreal
Alytes obstetricans	0	0	0
Discoglosus pictus	0	0	0
Barbourula busuanguensis	0	2	1
Bombina maxima	0	2	0
Xenopus clivii	0	2	1
Xenopus borealis	0	2	1
Spea multiplicata	0	0	0
Scaphiopus holbrookii	0	2	0
Xenophrys major	0	0	1
Brachytarsophrys carinensis	0	0	0
Leptobranchium hasseltii	1	0	0
Pelobates varaldii	0	0	0
Pelobates fuscus	0	2	0
Pelodytes caucasicus	0	2	0
Ascaphus truei	0	2	1
Ascaphus montanus	0	2	1
Leiopelma hamiltoni	0	2	0
Heleophrynae natalensis	1	1	1
Limnodynastes dorsalis	0	0	0
Crinia signifera	1	0	0
Mixophyes balbus	0	0	0
Rhinoderma darwini	0	1	0
Eusophus roseus	0	0	0
Crossodactylus trachystomus	1	0	0
Crossodactylus gaudichaudii	1	0	0
Batrachyla taeniata	0	0	0
Hylorina sylvatica	0	0	0
Zachaenus parvulus	1	0	0
Thoropa miliaris	0	0	0
Cycloramphus asper	1	0	0
Telmatobius	0	1	1

thompsoni			
Lepidobatrachus laevis	0	0	0
Ceratophrys aurita	0	0	0
Allophryne rutveni	0	0	2
Hyalinobatrachium fleischmanni	0	0	2
Centrolene buckleyi	0	0	2
Cryptobatrachus boulengeri	0	0	2
Gastrotheca cornuta	0	0	2
Gastrotheca peruana	0	0	2
Stefania scalae	0	0	2
Hemiphractus fasciatus	0	0	2
Hemiphractus proboscideus	0	0	2
Hemiphractus helioi	0	0	2
Hyla chrysoscelis	0	1	2
Hyla cinerea	0	1	2
Hyla femoralis	0	1	2
Hyla miliaria	0	1	2
Hyla mixomaculata	0	0	2
Hyla nephila	0	0	2
Hyla salvage	0	0	2
Nyctimantis rugiceps	0	0	2
Hyla smithi	0	0	2
Hyla velancifer	0	0	2
Boana boans	0	0	2
Acrys crepitans	0	0	2
Acrys gryllus	0	0	2
Anotheca spinosa	0	1	2
Aparasphenodon brunoi	0	1	2
Argenteohyla siemersi	0	1	2
Corythomantis greenngi	0	1	2
Hyla andersonii	0	1	2
Litoria aurea	0	0	2
Phyllobates bicolor	1	0	0
Silverstoneia flotator	1	0	0
Ameerega	1	0	0

trivittatus			
Colostethus latinasus	1	0	0
Minyobates minutus	0	1	0
Oophaga pumilio	1	0	0
Brachycephalus curupira	1	0	0
Brachycephalus ephippium	1	0	0
Ischnocnema quixensis	1	0	0
Ischnocnema gualteri	1	0	0
Barycholos pulcher	1	0	0
Craugastor laticeps	1	0	0
Odontophrynus americanus	1	0	0
Procerathophrys bioei	0	0	0
Macrogeniaglottus alipioi	0	0	0
Adenomera andreae	1	0	0
Anaxyrus terrestris	1	0	0
Anaxyrus fowleri	1	0	0
Adenomus kelaartii	1	0	0/2
Leptodactylus pentadactylus	1	0	0
Melanophryniscus stelzneri	1	0	0
Anaxyrus quercicus	1	0	0
Ansonia mcgregori	1	0	0
Atelopus oxyrhinchus	1	0	0
Atelopus ignescens	1	0	0
Duttaphrynus dodsoni	1	0	0
Frostius pernambucensis	1	1	2
Laurentophryne parkeri	1	0	0
Mertensophryne micronotis	1	0	0
Peltophryne guentheri	1	0	0
Truebella tothastes	1	0	0
Truebella skoptes	1	0	0
Capensibufo sp.	1	0	0

Didynamipus sjostedti	1	0	0
Rhinella dorbigni	1	0	0
Rhinella dyptica	1	0	0
Rhinella arenarum	1	0	0
Rhinella margaritifera	1	0	0/2
Nasikabatrachus sahyadrensis	0	1	0
Sooglosus gardineri	0	1	0
Sooglosus sechellensis	0	1	0
Balebreviceps hillmani	0	2	0
Callulina kisiwamsitu	0	2	0
Probreviceps macrodactylus	0	0	0
Spelaeophryne methneri	0	0	0
Hemisus guineensis	1	1	0
Kassinula wittei	0	2	2
Nyctibates corrugatus	0	0	2
Acanthixalus spinosus	0	0	2
Astylosternus diadematus	0	2	0
Arthroleptis bioko	0	2	0
Cardioglossa cyaneospila	0	0	0
Leptodactylodon ovatus	0	1	0
Trichobatrachus robustus	0	1	0
Leptopelis brevirostris	0	0	2
Leptopelis notatus	0	0	2
Otophrynae robusta	0	0	0
Paedophrynae amauensis	0	0	0
Synaptoranus mirandariberoi	0	1	0
Uperodon systoma	0	0	0
Syncope antenori	1	0	0/2
Melanobatrachus indicus	1	1	0

Chiasmocleis crucis	1	0	0
Gastrophryne carolinensis	1	1	0
Madecassophryne truebae	0	1	0
Odontobatrachus natator	0	1	1
Phrynobatrachus krefftii	0	0	2
Hildebrantia ornata	0	1	0
Ptychadena oxyrhynchus	0	1	0
Pyxicephalus adspersus	0	1	0
Tomopterna delandii	0	1	0
Arthroleptella lightfooti	0	0	0
Cacosternum namaquense	1	0	0
Cacosternum boettgeri	1	0	0
Microbatrachella capensis	0	1	0
Natalobatrachus bonebergi	0	1	2
Conraua goliath	0	0	1
Conraua crassipes	0	1	1
Petropedetes cf.vulpiae	0	0	2
Petropedetes sp.	0	0	2
Cerathobatrachus guentheri	0	2	2
Cornufer guppyi	0	2	0
Fejervarya limnocharis	0	2	0
Limnonectes macrocephalus	0	1	0
Limnonectes blythii	0	2	0
Nyctibatrachus cf. aliciae	0	0	0
Nyctibatrachus major	0	0	0
Rana capito	0	1	0
Rana leptodctyla	0	1	0
Lithobates clamitans	0	1	0

Occidozyga laevis	0	1	0
Amnirana albolabris	0	0	2
Indirana phrynoderma	0	0	0
Boophis boehmei	0	0	2
Buergeria japonica	0	1	2
Nyctixalus spinosus	0	2	2
Nyctixalus pictus	0	2	2
Philautus worcesteri	0	2	2
Philautus surdus	0	2	2
Philautus petersi	0	2	2
Polypedates nigripunctatus	0	0	2
Polypedates macrotis	0	0	2
Raorchestes brimarrumpfi	0	0	2
Raorchestes resplendens	0	0	0
Rhacophorus catamitus	0	0	2

Character matrix used in Mesquite 2.7 (Maddison and Maddison, 2019) through character mapping investigate the relationship between the palmar sesamoid, the carpal ventral surface, phylogeny, and the habitat use within the anuran groups.
