# Supplementary material for: An overview of the osseous palmar sesamoid in Anura, with the particular case of some Rhinella species
Source: PeerJ. 2023 May 15;11:e15063. doi: 10.7717/peerj.15063 (PMC10194070; doi:10.7717/peerj.15063)
Supplement: Supplemental Information 4 — We selected those species that have a bony palmar sesamoid and described it and the distal carpal anatomical context. Some details were included to extend the usefulness of our data to anatomists working in other topics. [file peerj-11-15063-s004.pdf]

Family	Taxon	Sesamoid	Carpals anatomy	Morphosource data
**Alytidae**	*Leiopelma hamiltoni*	Absent. There is a small sesamoid on the palmar surface of the manus, but located close to the metacarpal bones. It is not homologous to the palmar sesamoid.	Distal carpals seem to be absent, probably not mineralized (Stephenson, 1960), also the prepollex. There is a soft concavity on the ventral face of distal carpal 5, located distal to the ulnare. Ulnare squared, without process.	M13874-24295
**Megophryidae**	*Leptobrachium hasseltii*	There is a palmar sesamoid flat and rounded, located over and between the protruded ventral faces of the distal carpal 3+4, and of the distal carpal 5.	Distal carpal 3+4 is a large bone, also distal carpal 5. Each bone has a ventral protuberance, and together form a concavity that supports the sesamoid. Distal carpal 2 is small and rounded. The palmar carpal surface is concave in general (Fig. 2B).	M10832-16286

**Heleophryne**	*Hadromophryne natalensis*	The palmar sesamoid is elongated, oval and flat. Located oblique over the distal carpals 3, and 4+5.	The carpal distal 2 is cuboidal. Distal carpal 3, and carpal 4+5 with protuberances, forming a soft concavity where the palmar sesamoid fits. The ulnare is big, triangular, with a lateral crest. Radiale is also big and cuboidal (Fig. 2D). There is a hole on the medial side of the distal carpal 3+4+5, at the base of digit III.	M9316-12695
Myobatrachidae	*Crinia signifera*	This specimen exhibits a small and rounded palmar sesamoid, floating on the distal carpal 3+4+5 concavity. Only in the left manus.	The distal carpal 3+4+5 shows two parallel protuberances, forming a concavity in the palmar surface. The distal carpal 2 is small and rounded and located at the base of metacarpals 2 and 3.	M64730-116392

Hylodidae	*Crossodactylus trachystomus*	A large, flat, and oval palmar sesamoid is present, covering the depression of the distal carpal 3+4+5 concavity.	The distal carpal 3 +4+5 has two well-marked bulges, like crests, forming a concavity where the sesamoid fits. The distal carpal 2 is elongated, located at the large base of the metacarpus 2.	M13733-24014
	Crossodactylus gaudichaudii	A large, flat, and oval palmar sesamoid is present, covering the depression of the distal carpal 3+4+5 concavity.	The distal carpal 3+4+5 protrudes with a soft concavity where the sesamoid fits. Distal carpal 2 is large and rounded. There is a small hole in the medial side of the distal carpal 3+4+5, at the base of digit III.	M20787-39539
Dendrobatidae	*Ameerega trivittata*	Palmar sesamoid oval and large, covering the soft concavity of the protruded distal carpal.	Distal carpal 3+4+5 with two protuberances that form the concavity where the palmar sesamoid is housed.	M15692-29052

Colostethus latinasus	Similar to *Ameerega*. Palmar sesamoid, oval and large, fits into the concavity of the distal carpal 3+4+5, adjoining the crest of the ulnare	Distal carpal 3+4+5 with two parallel crests that form the concavity where the palmar sesamoid is housed.	M64056-115564
Oophaga pumilio	There is a flat, large, and triangular palmar sesamoid that fits partially into the concavity of the distal carpal 3+4+5.	The distal carpal 3+4+5, with two crests that form the concavity where the palmar sesamoid is housed. There is a hole in the medial side of the distal carpal 3+4+5, at the base of digit III.	M15470-28400
Phyllobates bicolor	There is a flat and oval palmar sesamoid lying over the concavity formed by two parallel crests of the distal carpal 3+4+5.	The distal carpal 3+4+5, with two crests that form the concavity where the palmar sesamoid is housed. There is a hole in the medial side of the distal carpal 3+4+5, at the base of digit III. Element Y is large	M15469-28391

	Silverstoneia flotator	There is a flat and oval palmar sesamoid lying over the concavity of the distal carpal 3+4+5.	The distal carpal 3+4+5 has two parallel crests that forma soft concavity where the palmar sesamoid is housed. There is a hole on the medial side of the distal carpal 3+4+5, at the base of digit III.	M63791-115217
Brachycephalidae	*Brachycephalus albolineatus*	The palmar sesamoid is located parallel to digit IV, elongated and small, lying over a soft concavity of the distal carpal 3+4+5.	The distal carpal 3+4+5 is protruded, and presents two small parallel crests forming a soft concavity where the sesamoid is housed. There is a hole on the medial side of the distal carpal 3+4+5, at the base of digit III.There is a large hole in the medial side of the distal carpal 3+4+5, at the base of digit III.	MHNCI 10295M000014739

Brachycephalus coloratus	The palmar sesamoid is located parallel to digit IV, elongated and small, lying over a soft concavity of the distal carpal 3+4+5.	The distal carpal 3+4+5 is protruded, and presents two small parallel crests forming a soft concavity where the sesamoid is housed. There is a hole on the medial side of the distal carpal 3+4+5, at the base of digit III.There is a large hole in the medial side of the distal carpal 3+4+5, at the base of digit III.	MHNCI 10274M000014739
Brachycephalus curupira	Small elongated, and pyramidal palmar sesamoid lying over a soft concavity of the distal carpal 3+4+5.	The distal carpal 3+4+5 is protruded and presents two soft parallel crests forming a concavity. There is a large hole in the medial side of the distal carpal 3+4+5, at the base of digit III.	M10229-14782
Brachycephalus ephippium	The palmar sesamoid and a concavity in the palmar surface of the manus are present.	The distal carpal 3+4+5 is protruded and presents two soft parallel crests.	M9209-12426

	Ischnocnema gualteri	The palmar sesamoid is elongated, large, and flat. It fits into the concavity formed by the two crests of the carpal 3+4+5.	The distal carpal 3+4+5 is large, protruded, and presents two parallel protuberances/crests forming a concavity. There is a large hole in the medial side of the distal carpal 3+4+5, at the base of digit III.	M13015-22193
	Ischnocnema quixensis	The palmar sesamoid is large, oval, and flat. It fits into the soft concavity between the two protuberances/crests of the carpal 3+4+5.	The distal carpal 3+4+5 is large, protruded, and presents two parallel protuberances/crests forming a concavity. There is a large hole in the medial side of the distal carpal 3+4+5, at the base of digit III	M13245-22890
Craugastoridae	*Craugastor laticeps*	There is a palmar sesamoid triangular, flat, and large that fits in a soft and wide concavity of the carpal 3+4+5.	The distal carpal 3+4+5 is large, protruded, and presents two parallel protuberances forming a wide and soft concavity. There is a hole in the medial side of the distal carpal 3+4+5, at the base of digit III.	M9311-12670

Strabomantidae	*Barycholos pulcher*	A large, flat, and elongated palmar sesamoid is visible. But the carpus is destroyed.	The carpus is destroyed	M12275-20040
	Barycholos pulcher	A large, oval, and elongated palmar sesamoid with a distal rounded head fits between the two parallel crests of the distal carpal 3+4+5. The proximal thinner part of the drop-shaped palmar sesamoid lies on the palm of the manus.	The distal carpal 3+4+5 is large, protruded, and presents two parallel crests forming a concavity. There is a small hole in the medial side of the distal carpal 3+4+5, at the base of digit III.	M14710-26382
Odontophrynidae	*Odontophrynus americanus*	There is a small oval, and elongated palmar sesamoid lying over a wide concavity of the distal carpal 3+4+5, between its two crests.	Distal carpal 3+4+5 has a wide concavity limited by two parallel crests. The ulnare and radiale are big and cuboidal bones. The element Y is also big. The distal carpal 2 is small.	M25346

Leptodactylidae	*Adenomera andreae*	There is a large, rectangular, and elongated palmar sesamoid, that fits partially in the concavity of the distal carpal 3+4+5, between the two parallel crests.	Distal carpal 3+4+5 has two parallel crests that form a deep concavity. There is an elongated and rounded distal carpal 2. The element Y is also elongated and articulates with a two element formed prepollex. The ulnare bone has a ventral crest.	M9784-13750
Bufonidae	*Adenomus kelaartii*	A large and elongated palmar sesamoid with a rounded, wide distal extreme, that fits between the two parallel crests of the distal carpal 3+4+5. The proximal extreme is narrower and covers the palmar face of the manus. This form gives the palmar sesamoid a drop-shape.	The distal carpal 3+4+5 presents a narrow concavity limited by two parallel crests. Only the distal portion of the palmar sesamoid is over it. There is a hole in the medial side of the distal carpal 3+4+5, at the base of digit III.	M14189-24953

Anaxyrus fowleri	A large and elongated palmar sesamoid with a rounded, wide distal extreme, that fits between the two parallel crests of the distal carpal 3+4+5. The proximal extreme is narrower and covers the palmar face of the manus. This form gives the palmar sesamoid a drop-shape.	The distal carpal 3+4+5 presents a concavity limited by two parallel crests.	M8870-11366
Anaxyrus quercicus	A large and elongated palmar sesamoid with a rounded, wide distal extreme, that fits between the two parallel crests of the distal carpal 3+4+5. The proximal extreme is narrower and covers the palmar face of the manus. This form gives the palmar sesamoid a drop-shape.	The distal carpal 3+4+5 presents a concavity limited by two parallel crests.	M8871-11372

Anaxyrus terrestris	A large, oval, and elongated palmar sesamoid with a rounded, wide distal extreme, that fits between the two parallel crests of the distal carpal 3+4+5. The proximal extreme is narrower and covers the palmar face of the manus. This form gives the palmar sesamoid a drop-shape.	The distal carpal 3+4+5 presents a concavity but only the distal part of the palmar sesamoid is over it. There is a hole in the distal carpal 3+4+5, at the base of digit 3, is present and small.	M8872-11374
Ansonia mcgregori	A small and oval palmar sesamoid fits into a smooth concavity, between the two parallel crests of the distal carpal 3+4+5	Distal carpal 3+4+5 two parallel crests forming a concavity where palmar sesamoid fits. The hole in the distal carpal 3+4+5, at the base of digit 3, is present.<br><br>.	M14201-24984

Atelopus ignescens	A oval and small palmar sesamoid fits into a smooth concavity, between the two parallel crests of the distal carpal 3+4+5	Distal carpal 3+4+5 with a big protuberance. A big hole is visible in this protuberance, at the level of the base of digit III. Ulnare is cuboidal and big. A big and irregular element Y is evident.	M8902-11496
Atelopus oxyrhynchus	A small and oval palmar sesamoid fits into the soft concavity, formed by the two parallel crests of the distal carpal 3+4+5.	Distal carpal 3+4+5 with a big protuberance. A big hole is visible in this protuberance, at the level of the base of digit III.	M8869-11363

Capensibufo sp.	A large and elongated palmar sesamoid with a distal rounded head over the concavity between the two parallel crests of the distal carpal 3+4+5. The proximal extreme is narrower and extends over the palmar face of the manus. This form gives the palmar sesamoid an almost rectangular shape.	The distal carpal 3+4+5 presents a soft concavity limited by two parallel crests. The ulnare presents a ventral apophysis. The radiale is partially covered by the palmar sesamoid and articulates with the element Y.	M14385-25498
Duttaphrynus dodsoni	A large and elongated palmar sesamoid with a distal rounded head over the concavity between the two parallel crests of the distal carpal 3+4+5. The proximal extreme is narrower and extends over the palmar face of the manus. This form gives the palmar sesamoid an almost cone shape.	The distal carpal 3+4+5 presents a soft concavity limited by two low parallel crests. A small hole is evident at the base of digit III. The ulnare presents a ventral apophysis. The radiale is partially covered by the palmar sesamoid and articulates with the element Y.	M14411-25585

Didynamipus sjostedti	An elongated and oval palmar sesamoid fits into the two parallel crests of the distal carpal 3+4+5.	Distal carpal 3+4+5 has two protuberances/crests that form a narrow concavity where the sesamoid is partially located.	M13311-23005
Frostius pernambucensis	The oval palmar sesamoid fits into the concavity of a ventrally protruded distal carpal 3+4+5.	Distal carpal 3+4+5 has a narrow concavity where the sesamoid is partially located. There is a large hole that faces the digit III, in the distal carpal.	M17368-32526
Laurentophryne parkeri	There is a rounded sesamoid that fits into the soft concavity formed by two parallel crests of the distal carpal 3+4+5.	Distal carpal 3+4+5 has a narrow concavity where the sesamoid is partially located. There is an oval hole in the distal carpal that faces the digit III. The ulnare has a ventral crest.	M17026-31690

Melanophryniscus stelzneri	This specimen presents two palmar overlapped oval sesamoids, one on top of the other, that fits into the two parallel crests of the distal carpal 3+4+5.	The distal carpal 3+4+5 has a narrow concavity limited by the two crests (Fig. 2E). There is an oval and large hole in the distal carpal that faces to the digit III.	M9213-12431
Mertensophryne micranotis	A big and rounded palmar sesamoid fits into the concavity formed by two parallel crests of the distal carpal 3+4+5.	The concavity formed by two parallel crests of the distal carpal 3+4+5 is narrow.	M14412-25588
Peltophryne guentheri	A large and triangular palmar sesamoid with a distal part over the concavity of the distal carpal 3+4+5. The proximal extreme is narrower and extends over the palmar face of the manus. This form gives the palmar sesamoid a triangle shape.	Distal carpal 3+4+5 with a wide concavity limited by the two parallel crests.	M8873-11379

	Truebella skoptes	Small and drop-shaped palmar sesamoid that fits into the cavity of the distal carpal 3+4+5	Distal carpal 3+4+5 with a narrow concavity limited by the two parallel crests. There is an oval and large hole in the distal carpal that faces to the digit III.	M20818-39601
	Truebella tothastes	Small and oval palmar sesamoid that fits into the two parallel crests of the distal carpal 3+4+5. Only evident in the left manus.	Distal carpal 3+4+5 with a concavity limited by the two parallel crests.	M20817-39599
Cycloramphidae	*Cycloramphus asper*	Rounded and flat palmar sesamoid fits into the soft concavity of the distal carpal 3+4+5.	Distal carpal 3+4+5 has a soft concavity limited by two parallel crests	M13198-22882

	Zachaenus parvulus	Rounded and flat palmar sesamoid fits into the concavity of the distal carpal 3+4+5.	Distal carpal 3+4+5 is small and protruded to the ventral surface of the manus. It has a concavity formed by two parallel crests that partially house the palmar sesamoid.	M13305-23008
**Microhylidae**	*Syncope antenori*	The small and rounded palmar sesamoid is housed in the concavity of the distal carpal 3+4+5.	Distal carpal 3+4+5 is protruded to the ventral surface of the manus. It has a wide concavity formed by two parallel crests that houses the palmar sesamoid.	M11048-16850
	Melanobatrachus indicus	A flat, oval and ventrally curved sesamoid fits into the distal carpal 3+4+5 concavity.	Distal carpal 3+4+5 is protruded to the ventral surface of the manus. It has a narrow concavity formed by two parallel crests where the palmar sesamoid fits.	M12125-19810

	Chiasmocleis crucis	A flat, irregular-shaped and ventrally curved sesamoid fits into the distal carpal 3+4+5 concavity.	Distal carpal 3+4+5 has a concavity formed by two parallel crests where the palmar sesamoid fits.	M13915-24429
	Gastrophryne carolinensis	A small and elongated palmar sesamoid located over the soft concavity of the distal carpal 3+4+5.	Distal carpal 3+4+5 has a wide and soft concavity limited by two parallel crests.	M12302-20081
**Hemisotidae**	*Hemisus guineensis*	A large, square, and flat palmar sesamoid that lies on the protruded ventral surface of the distal carpal 3+4+5 (Fig. 2 A).	Distal carpal 3+4+5 has a wide and soft concavity on its ventral surface. No crests/protuberances are evident.	M15807

**Pyxicephalidae**	*Cacosternum boettgeri*	A small and oval palmar sesamoid, fits into the concavity of distal carpal 3+4+5.	Distal carpal 3+4+5 has a narrow concavity limited by two parallel crests that house the palmar sesamoid. A small hole is evident at the base of digit III	M13936-24502
	Cacosternum namaquensis	An oval palmar sesamoid, fits into the two protruded crests of the distal carpal 3+4+5.	Distal carpal 3+4+5 has a narrow concavity limited by two parallel crests that house the palmar sesamoid. A small hole is evident at the base of digit III	M13937-25450

SM4. Morphological descriptions of the ventral surface of the manus of species downloaded from Morphosource CT imagens. We selected those species that have a bony palmar sesamoid and described it and the distal carpal anatomical context. Some details were included to extend the usefulness of our data to anatomists working in other topics.
