# Supplementary figures and images for: An overview of the osseous palmar sesamoid in Anura, with the particular case of some Rhinella species
Source: PeerJ. 2023 May 15;11:e15063. doi: 10.7717/peerj.15063 (PMC10194070; doi:10.7717/peerj.15063)

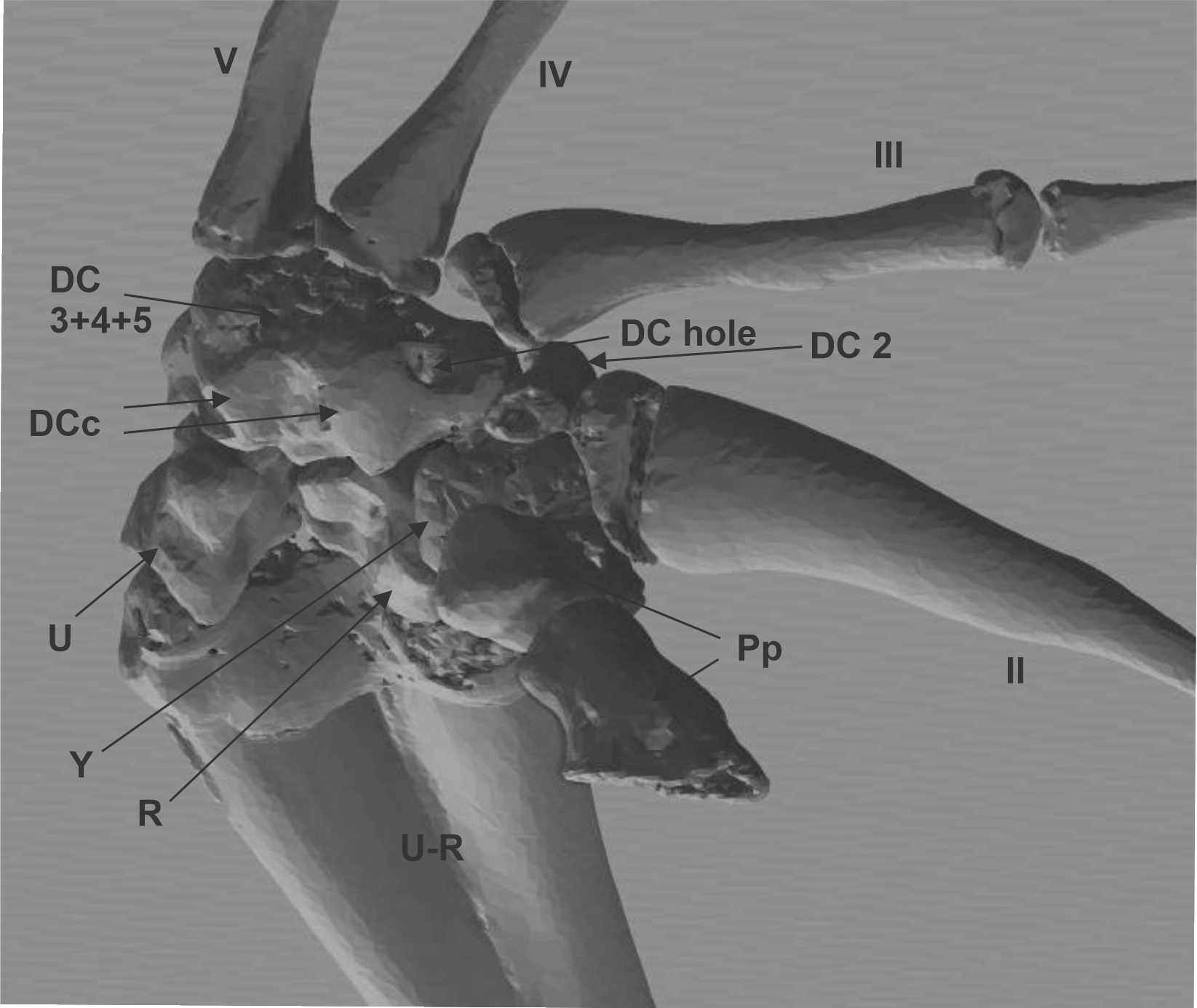

Supplement: Supplemental Information 5 — The muscles, flexor plate and the sesamoid were removed to show the distal carpal 3+4+5 form. DC distal carpal 3+4+5; DCc distal carpal 3+4+5 parallel crests; DC hole distal carpal 3+4+5 hole; DC2 distal carpal 2; U ulnar; R radial; U-R radio-ulna; Pp prepollex; Y Y element. Digits are enunciated in roman notation as II, III, IV, and V. [file peerj-11-15063-s005.png]
